# Genetic Structure of Two Protist Species (Myxogastria, Amoebozoa) Suggests Asexual Reproduction in Sexual Amoebae

**DOI:** 10.1371/journal.pone.0022872

**Published:** 2011-08-01

**Authors:** Anna Maria Fiore-Donno, Yuri K. Novozhilov, Marianne Meyer, Martin Schnittler

**Affiliations:** 1 University of Greifswald, Institute of Botany and Landscape Ecology, Greifswald, Germany; 2 V. L. Komarov Institute of Botany, Russian Academy of Sciences, Laboratory for Systematics and Geography of Fungi, St. Petersburg, Russia; 3 Le Bayet, Rognaix, France; Université de Genève, Switzerland

## Abstract

Plasmodial slime molds (Myxogastria or Myxomycetes) are common and widespread unicellular organisms that are commonly assumed to have a sexual life cycle culminating with the formation of often macroscopic fruiting bodies that efficiently disseminate spores. However, laboratory studies based on mating compatibility revealed the coexistence of asexual as well as sexual strains. To test this hypothesis in natural populations, we investigated the genetic variability of two species of the genus *Lamproderma*. Detailed ecological relevés were carried out in 2007 and 2009 in several deep ravines in the Elbsandsteingebirge (Saxony, south-eastern Germany). Morphological characters of 93 specimens of *Lamproderma* were recorded and genetic analyses, based on the small subunit ribosomal gene, the internal transcribed spacer 1 and partial elongation factor 1α sequences were carried out for 52 specimens. Genetic analyses showed the existence of two major clades, each composed of several discrete lineages. Most of these lineages were composed of several identical sequences (SSU, ITS 1 and EF-1α) which is explained best by an asexual mode of reproduction. Detrended Correspondence Analysis of morphological characters revealed two morphospecies that corresponded to the two major clades, except for one genotype (Lc6), thus challenging the morphospecies concept. Genetic patterns were not related to the geographical distribution: specimens belonging to the same genotype were found in distinct ravines, suggesting effective long-distance dispersal via spores, except for the Lc6 genotype which was found only in one ravine. Implications for the morphological and biological species concept are discussed.

## Introduction

Why is sex predominant in higher eukaryotes? The paradox of the maintenance of sex arises from the fact that it has many immediate costs, compared to the advantages given by asexual reproduction [1], [2]. Recent models that include *facultative* sex – that is, asexual reproduction with infrequent rounds of sex [3], as it is encountered in most protists [4], still conclude that sex is evolutionary favorable (see [5] and references therein). Current models on evolution of sexuality are based largely on multicellular organisms such as plants, fungi and metazoans, which represent only a very small fraction of the genetic diversity of eukaryotes – most to be found in various high-ranking clades of protists [6], [7], [8]. To shed some light on this question, we investigated the genetic structure of a group of protists, the plasmodial slime molds (Myxogastria, also called Myxomycetes). Myxogastria present unique advantages for studying factors shaping the proportion of sexual/asexual strains in nature. Sporocarps can be stored for decades, and the very resistant spores are reservoirs of DNA for molecular studies [9], [10]. If sex (and thus meiosis) occurs, the spores should represent the offspring of two parents and the genes found in the sporocarps originating from a single plasmodium will show a mixture of the original alleles from both parental amoebae as well as their recombination products. If the sporocarps are produced asexually, no major variation is expected.

Myxogastria form a monophyletic taxon in the phylum Amoebozoa [11], [12] together with the better known *Entamoeba*, *Acanthamoeba* and the model organism *Dictyostelium discoideum* [13], [14], [15], [16]. Myxogastria include the model organism *Physarum polycephalum*, whose genome is currently being completed [17], [18]. Recently it has been found that myxogastrians are one of the major components of protist soil biodiversity [19]. While in most amoebozoans sexuality has never been reported, the recently recognized clade called “macromycetozoa” composed of Dictyostelia, *Ceratiomyxa* and Myxogastria [14] is characterized by alternating asexual and sexual reproduction. Most members of this clade form macroscopic fruiting bodies that efficiently disperse spores [20]. The distinctive fruiting bodies of Myxogastria have been intensely collected worldwide [21] and ca. 900 morphospecies are recognized [22]. Myxogastria have a complex life cycle with alternating sexual and asexual, haploid and diploid stages [20]. Asexual reproduction in Myxomycetes is of two very different kinds: the clonal reproduction of haploid amoebae preceding the sexual part of the life cycle, and variants of the originally sexual life cycle, producing spores with no genetic variation (Table 1). The sexual stage starts when two compatible amoebae fuse to form a diploid zygote, which becomes a plasmodium of often macroscopic dimensions that will give rise to the fruiting bodies containing spores. Meiosis is assumed to occur during spore formation; spores should thus be haploid and represent a new generation.

**Table 1:** Hypothetical reproductive systems in Myxogastria and their observable consequences.

	Asexuality		Sexuality
**Mating system**	Non-heterothallic	Homothallic	Heterothallic
	Truly asexual Apomictic	No mating-types Cryptic sexuality	Mating types
**Spores & amoebae**	1n or 2n throughout	1n	1n
**Zygote**	None	Formed by fusion of two amoebae	
**Change in ploidy between amoebae and plasmodium**	None	Yes	Yes
**Homo- (Ho) or heterozygocity (He)**	If 2n: Ho (rarely He?)	Ho	Ho or He
**Meiosis**	None	Yes	Yes
**Recombination**	None	Not effective	Yes
**Spores (offspring)**	All identical to the parental type^1^	All identical to the parental type^1^	Genetic diversity Two parental types & recombinants
**Single-spore cultures produce sporocarps**	Yes	Yes	No
**Distinct alleles**	No^1^	No^1^	Yes
**SNP's likely to occur**	No^1^	No^1^	Yes
**Genetic exchange between strains**	No	No	Yes
**Genetic structure of the species**	Distinct clones	Distinct clones	Genetic diversity

1 Possible rare mutations not considered.

What is known of Myxogastria sexuality stems mostly from culture-based studies conducted from the sixties to the nineties [23], [24], based on compatibility tests between strains, and therefore limited to a minority of species (mostly Physariida) that can be isolated. Nevertheless, the picture from these pioneering studies indicates that each morphospecies could be composed of an intricate pattern of core sexual strains, surrounded by a swarm of asexually reproducing clones that differ genetically and may differ in observable morphological traits (see [25] and references therein; [26]).

Heterothallism (sexual cycle including fusion of two distinct amoebae) is most often achieved via a single-locus multiallelic system, like in *Didymium iridis* [27], [28], or by two loci with multiple alleles, as demonstrated for *Physarum polycephalum* [29]. Strains that can form fruiting bodies from single-spore cultures are called non-heterothallic, since there are two conceivable reproductive options. First, genetically identical amoebae could mate resulting in a diploid homozygous plasmodium (due to a breakdown of the mating system). This cryptic sexual cycle involves two identical genomes, but some variability could be generated if mutations have occurred. Second, strains can be truly asexual (apomictic) and form plasmodia without mating. Such apomictic strains could be haploid or diploid during the entire cycle without any change in the nuclear content and, if diploid, could be homozygous or heterozygous, depending on the state of the sexual ancestors (Table 1). Whether asexual lineages are truly apomictic or have a cryptic sexuality is still unclear, but some lines of evidence point towards true apomixis: isolates usually do not crossbreed [27] and measurements of DNA contents in myxamoebae and plasmodia are identical and appear to be 2n [30]. Cryptic sexuality may thus be only a hypothetical option that could not be ruled out by culture-based studies.

Taking advantage of previous phylogenetic studies [31] and numerous collections [32], we targeted the genus *Lamproderma*, which comprises 48 species [33], displaying the typical characteristics of the order Stemonitida: dark spores, internal secreted stalks and a transparent to white plasmodium. In addition, all species are characterized by a persistent, often iridescent peridium (a sheath surrounding the spore mass). This study focuses on *Lamproderma* species restricted to cool and very humid microclimates, fruiting on moss- and algae-covered wood and rocks and therefore called bryophilous [34], [35]. In this ecological guild, three *Lamproderma* species are currently described [36]: *Lamproderma columbinum*, widespread in temperate zones, *L. puncticulatum* with a more restricted distribution [37], and *L. granulosum*, only described from that region and not known elsewhere [38] (Supporting information S1, S2, S3). We collected these species in the very peculiar habitat of deep and narrow sandstone ravines in Saxony (south of Dresden, Germany) and determined them according to fruiting body morphology [33], [36]. A previous detailed study in the same localities has shown that: 1) statistical analyses of morphological characters clearly separate *L. columbinum* from *L. puncticulatum*; 2) *L. puncticulatum* is composed of two groups, differing by spore size and ornamentation; 3) *L. granulosum* may be conspecific with *L. puncticulatum* [32].

The goals of this study are: 1) assessing the reproductive mode in the morphospecies of *L. columbinum* and *L. puncticulatum*; 2) searching for concordance between the morphological traits that determine species and molecular markers; 3) estimating dispersal and gene flow among populations collected in a restricted area.

## Materials and Methods

### Field sampling and detailed relevés

Between Sept. 26 and Oct. 4, 2007 and Oct. 4 to 7, 2009, several ravines in the Saxonian Switzerland National Park (Saxony, Germany) were surveyed and specimens of bryophilous Myxogastria were systematically collected on bryophyte-covered boulders and rocks, yielding 93 *Lamproderma* spp. specimens from 12 localities. The 205 records (1–126 collected in 2007 and 127–205 in 2009) were associated with vegetation relevés and measurements of a set of environmental parameters. These ravines were characterized by a nearly constant temperature of 10±2°C (independent of season) and a constant humidity of 97–100%. Localities, methods and results of the 2007 relevés have been described in detail [32]. A list of the specimens used in this study (Table 2 and Supporting information S4) and a map showing the localities of collection is provided (Supporting information S5). For comparison, two additional specimens of *Lamproderma columbinum* (F1 and F2) were collected in France (Savoie and Haute-Savoie, Table 2).

**Table 2:** Source and identification of the 52 DNA samples used in this work.

Spe-cimen #	Loca-lities #	Mor-pho-type	Genetic group	Date of collection	Latitude (N)	Longi-tude (E)	Herba-rium #	GenBank Accession #		
								SSU	ITS 1	EF-1α
**2**	**3**	col	Lc2	27.09.2007	50.9686	14.0425	21775			
**3**	**3**	pun	Lp2	27.09.2007	50.9686	14.0425	21776	HQ687195		
**6**	**3**	pun	Lp3	27.09.2007	50.9686	14.0425	21779			JF431072
**7**	**3**	pun	Lp3	27.09.2007	50.9686	14.0425	21780			
**8**	**3**	pun	Lp3	27.09.2007	50.9686	14.0425	21781			
**30**	**5**	pun	Lp1	28.09.2007	50.9772	14.0389	21800		HQ692815	JF431070
**32b**	**5**	pun	Lc6	28.09.2007	50.9772	14.0389	21802			
**33**	**5**	pun	Lc6	28.09.2007	50.9772	14.0389	21803			
**50**	**5**	pun	Lp1	30.09.2007	50.9772	14.0389	21816			
**54**	**5**	pun	Lc6	30.09.2007	50.9772	14.0389	21822			
**55**	**5**	pun	Lc6	30.09.2007	50.9772	14.0389	21824			
**57**	**5**	col	Lc4	30.09.2007	50.9772	14.0389	21826			
**58**	**5**	pun	Lc6	30.09.2007	50.9772	14.0389	21828			
**63**	**5**	pun	Lc6	30.09.2007	50.9772	14.0389	21833			JF431068
**63b**	**5**	pun	Lc6	30.09.2007	50.9772	14.0389	AMFD268	HQ687197	HQ692812	
**64**	**6**	col	Lc2	30.09.2007	50.9753	14.0458	21834			
**73**	**10**	pun	Lp2	01.10.2007	50.9208	14.2889	21843		HQ692816	
**77**	**11**	pun	Lp1	01.10.2007	50.9014	14.2958	21847			
**90**	**9**	col	Lc1	03.10.2007	50.9186	14.1944	21860	HQ687198	HQ692808	
**91**	**9**	pun	Lp1	03.10.2007	50.9186	14.1944	21861			
**94**	**9**	col	Lc4	03.10.2007	50.9186	14.1944	21864	HQ687199		
**96**	**8**	col	Lc2	03.10.2007	50.9708	14.1033	21866			
**97**	**8**	col	Lc2	03.10.2007	50.9708	14.1033	21867			
**102**	**10**	pun	Lp2	04.10.2007	50.9208	14.2889	21872			
**106**	**10**	col	Lc2	04.10.2009	50.9208	14.2889	21876	HQ687196		JF431066
**123**	**12**	pun	Lp1	04.10.2009	50.8989	14.2958	21890			
**132**	**7**	col?	Lc7	03.10.2009	50.9703	14.1017	21898	HQ687200	HQ692813	JF431069
**137**	**12**	pun	Lp2	04.10.2009	50.8989	14.2958	21902			JF431071
**144**	**12**	col	Lc5	04.10.2009	50.8989	14.2958	21907	HQ687201		JF431067
**146**	**12**	pun	Lp1	04.10.2009	50.8989	14.2958	21909			
**152**	**11**	pun	Lp2	04.10.2009	50.9014	14.2958	21915			
**153**	**11**	pun	Lp2	04.10.2009	50.9014	14.2958	21916			
**154**	**11**	pun	Lp2	04.10.2009	50.9014	14.2958	21917			
**155**	**11**	col	Lc2	04.10.2009	50.9014	14.2958	21918			
**160**	**4**	pun	Lp3	05.10.2009	50.9750	14.0361	21921			
**162**	**4**	pun	Lp3	05.10.2009	50.9750	14.0361	21923	HQ687202	HQ692817	
**170**	**5**	pun	Lc6	05.10.2009	50.9772	14.0389	21929			
**171**	**5**	pun	Lc6	05.10.2009	50.9772	14.0389	21930			
**172**	**5**	pun	Lp1	05.10.2009	50.9772	14.0389	21931	HQ687194		
**173**	**5**	pun	Lc6	05.10.2009	50.9772	14.0389	21933			
**177**	**1**	col	Lc2	05.10.2009	50.9656	14.0319	21938			
**179**	**1**	col	Lc3	05.10.2009	50.9656	14.0319	21940	HQ687203	HQ692810	
**180**	**1**	col	Lc3	05.10.2009	50.9656	14.0319	21941			
**182**	**1**	col	Lc4	05.10.2009	50.9656	14.0319	21943			
**185**	**1**	col	Lc2	05.10.2009	50.9656	14.0319	21945			
**189**	**2**	pun	Lc4	07.10.2009	50.9661	14.0306	21949		HQ692811	
**192**	**2**	col	Lc2	07.10.2009	50.9661	14.0306	21953		HQ692809	
**198**	**2**	pun	Lp2	07.10.2009	50.9661	14.0306	21962			
**200**	**2**	col	Lc4	07.10.2009	50.9661	14.0306	21961			
**203**	**2**	pun	Lp3	07.10.2009	50.9661	14.0306	21965			
**F1**	n.a.	col	Lc8	16.11.2005	46.3006	6.2607	AMFD349		HQ692814	
**F2**	n.a.	col	Lc7	11.10.2007	45.5791	6.4248	MM37278	HQ687204		

Localities numbers in the Saxonian Switzerland region (SE of Dresden)“*Uttewalder Grund*” **1**: trail to “Teufelsschlüchte” 1.6 km N Stadt Wehlen; **2**: round trail “Teufelsschlüchte” 1.6 km N Stadt Wehlen; **3**: Lower “Zscherregrund” 1.3 km NNE Stadt Wehlen; **4**: between restaurant “Waldidyll” and “Felsentor” 1.8 km N Stadt Wehlen; **5**: near branch to “Bruno-Barthel-Weg” 1.9 km N Stadt Wehlen; **6**: “Kohlgrund”, upper part 1.7 km NNE Stadt Wehlen; “*Polenztal*” **7**: from “Neumühle” to “Waltersdorfer Mühle”, 1.5 km SSW Hohnstein; **8**: from “Annenloch” to “Waltersdorfer Mühle” 1.5 km SSW Hohnstein; “*Schrammsteine*” **9**: “Schießgrund” 1.3 km ESE Ostrau; “*Großer Zschand*” **10**: from “Neumannsmühle to branch to “Winterstein” 2.5 km S Ottendorf, **11**: “Richterschlüchte” 5 km S Ottendorf; **12**: “Weberschlüchte” 5.3 km S Ottendorf; *France* **F1**: France, Haute-Savoie, Chens-le-Pont; **F2**: France, Savoie, Tarentaise, Rognaix. Herbaria: MM = M. Meyer, AMFD = AM Fiore-Donno. Numbers starting with 21, M. Schnittler, deposited at Botanische Staatsammlung München.

### DNA extraction, amplification and sequencing

DNA was extracted from 5–6 adjacent sporocarps (most probably arising from a single plasmodium) using the DNeasy plant mini-kit (Qiagen, Hilden, Germany). Sporocarps were cooled to −80°C in a 2 ml safe-lock eppendorf tube with a single metallic bead of 5 mm diameter and then disrupted using a ball mill (Retsch MM301, 1 min, 30 Hz). We followed the manufacturers protocol except for the final step where DNA was eluted in 50 µl of the elution buffer (instead of 200 µl). Even so, the amount of DNA was undetectable by both electrophoresis and spectrophotometry. The first part of the SSU was amplified using the primers S2 [31] and SU19R (cgttaaagttgttgcggtta; all primers written as 5′ – 3′, with sequences in lower-case for reverse primers). For selected specimens, the complete SSU was amplified using already published primers [31]. ITS 1 primers were designed on the 3′ end of the SSU gene of our sequences and on the 5.8S of the few Myxogastria sequences publicly available. The pair SFB2 (GGTAATCGTAGGTGAACCTGCG) and 5.8RLam2 (cgataacgcttgtgactcgca) amplified the ITS 1 for all samples except for specimens of the groups Lc1, Lc2 and Lc3 (Table 2); here the primer pair SFBE (GAAGCAGAAGTCGTAACAAGGTA) and 5.8SR (ccgttaggcgatggattgyttgg) was used. The first part of the EF-1α gene was obtained using the primers 1FPhy (GCAAGTCCACCACCACTGG) and E800R [9], to obtain a ca. 700 bp long amplicon. Amplification parameters were adapted accordingly: elongation time depending on the length of the expected product (1–2 min) and hybridization temperature according to the primers (52–58°C). Cloning (when necessary) and sequencing were performed according to the manufacturer protocols (pGEM-T-Easy Vector System, Promega).

### Sequence alignments

To assess the phylogenetic position of the *Lamproderma* spp. collected in Saxony, we obtained nine nearly complete SSU rRNA gene sequences (1811–1890 bp excluding introns). We assembled them with those of selected representative of Stemonitida according to current phylogenies of Myxogastria [14], [31], [39]. Sequences that were identical (or nearly so) or partial sequences in clades already well represented were not included. An alignment of 1598 positions, comprising 21 taxa, was made using BioEdit version 7.0.9 [40] and used for phylogenetic analyses (Supporting information S6).

To assess the genetic structure of populations of *Lamproderma* spp. collected in Saxony, we obtained partial SSU rRNA gene sequences (the first ca. 650 nucleotides) from 52 samples. This fragment includes three variable regions and eleven helices that vary both in length and sequence: 6, 9, 10, E10_1 and 11 to 17 [41]. For example, the variable region I (helix 6) ranges from 31 to 39 bp. Particular attention was given to the secondary structure of these variable regions, which was inferred using *Physarum polycephalum* as a template [42]. Alignments of the helices were checked using the RNAalifold web server (http://rna.tbi.univie.ac.at/cgi-bin/RNAalifold.cgi, last accessed 10.10.2010). Sequences were either strikingly different (maximum 73% of similarities) or identical to each other. Only unique sequences, 11 in total, were used for subsequent analyses in a matrix of 498 unambiguously aligned sites (Supporting information S7), and deposited in the GenBank/EMBL database under accession numbers HQ687194–687204 (Table 2). To further investigate genetic relations inside clusters of identical sequences, we obtained 20 ITS 1 sequences. The length of the ITS 1 was so variable (from ca. 300 to 900 nucleotides) that no alignment was possible between clades. The analysis of the secondary structure showed some patterns common to each clade, but no pattern was common to all sequences, confirming that these ITS 1 sequences could not be used for phylogenetic analyses. The ten unique ITS 1 sequences are assembled in a file that is only partially aligned (Supporting information S8), and have been deposited in the GenBank/EMBL database under accession numbers HQ692808–692817 (Table 2). To further investigate the genetic variability we obtained 19 partial sequences of the elongation factor 1α (EF-1α). This fragment includes a very variable spliceosomal intron that seems to be obligatory in myxogastrians. The seven unique sequences have been deposited in GenBank under accession numbers JF431066–431072 (Table 2). The 19 newly obtained sequences are included in a partial EF-1α (ca. 1000 positions) myxogastrian alignment, showing the highly variable intron at position 692 (Supporting information S9). The alignment done for phylogenetic analyses of the 19 *Lamproderma* sequences comprised 573 nucleotide sites of which 69.5% were constant. An amino-acid alignment would have been too conserved for phylogenetic analyses, since there were only 11 variable sites.

### Phylogenetic analyses

Complete SSU rRNA gene sequences: The general time reversible model taking into account a gamma-distributed rate heterogeneity among sites (GTR+gamma) [43], [44] was selected using jModelTest 0.1.1 [44], [45]. Maximum likelihood (ML) analyses were run using RAxML 7.0.4 [46] with the GTR model of substitution and a 25 rate category discrete gamma distribution. The alignment comprised 522 patterns where the proportion of gaps and ambiguous nucleotides was negligible (0.012%). The best-scoring ML tree was inferred from 100 randomized starting Maximum Parsimony trees using the GTRMIX model (average log likelihood: −9065.271900, best log likelihood: −9065.271651). The best-scoring tree was used to report the confidence values as percentages obtained through 1000 non-parametric bootstraps under the GTRCAT model. Bayesian search of tree space was conducted using MrBayes, version 3.1.2 [47] with the GTR+gamma model of substitution, the gamma distribution being approximated by eight categories. Two runs starting from different random trees were performed and sampled every ten generations, with eight simultaneous chains, for one million generations. Convergence of the two runs, evaluated by a standard deviation of split frequencies <0.01, was reached after 18000 generations, and the 1800 trees obtained beforehand were discarded as burn-in. The remaining trees were assembled in a consensus tree (log likelihood = −9081.880, alpha = 0.234384).

The analysis of the partial SSU rRNA gene sequences was conducted as detailed above. The Maximum likelihood analyses determined that the alignment (498 positions, 11 taxa) comprised 111 patterns where the proportion of gaps and ambiguous nucleotides was negligible (0.0035%). The log likelihood of the best-scoring ML tree was −1746.132282. During Bayesian analyses, the convergence of the two runs was reached after 18000 generations (consensus tree: log likelihood = −1756.950; alpha = 0.266836). The star-tree was drawn using Phylowidget [48].

The Bayesian analysis of the 19 partial EF-1α sequences was conducted as indicated above, with the GTR+gamma model of substitution with four categories (jModelTest log likelihood of this model = −1199.64810). The convergence of the two runs was reached after 100000 generations and the first 10000 trees were discarded as burn-in (consensus tree: log likelihood = −1226.380; alpha = 22.875843).

### Mantel test

To test for correlation between geographic and genetic distances we performed a Mantel test. Genetic distances from the 498 bp alignment for the 50 specimens collected in Saxony were calculated using DNADIST v. 3.5c (implemented in BioEdit). Geographic coordinates were retrieved from a 1∶25000 topographic map, since GPS signals could not be received in the deep ravines. Adjacent relevés of one section of a ravine (usually 100–500 m in length) were grouped together; in total we calculated the distances between 12 localities (Table 2). A second matrix was made with the geographic distances between each of the 50 specimens. A Mantel test (999 iterations) was performed using PopTools v. 3.1.1 [49].

### Statistical evaluation of morphological characters

To resolve the taxonomy of the 93 collections of *Lamproderma* spp., 14 morphological characters were recorded, coded quantitatively and subjected to a correspondence analysis. We first used Principal Correspondence Analysis (PCA), but due to the unimodal distribution of samples over a gradient in trait expression, we also employed Detrended Correspondence Analysis (DCA) to remove possible arch and horseshoe effects [50]. The program PC-Ord 5.0 for Windows [51] was used; for DCA 15, 26 (default) and 50 segments were applied.

## Results

### Phylogenetic position of Lamproderma

**Figure 1:**
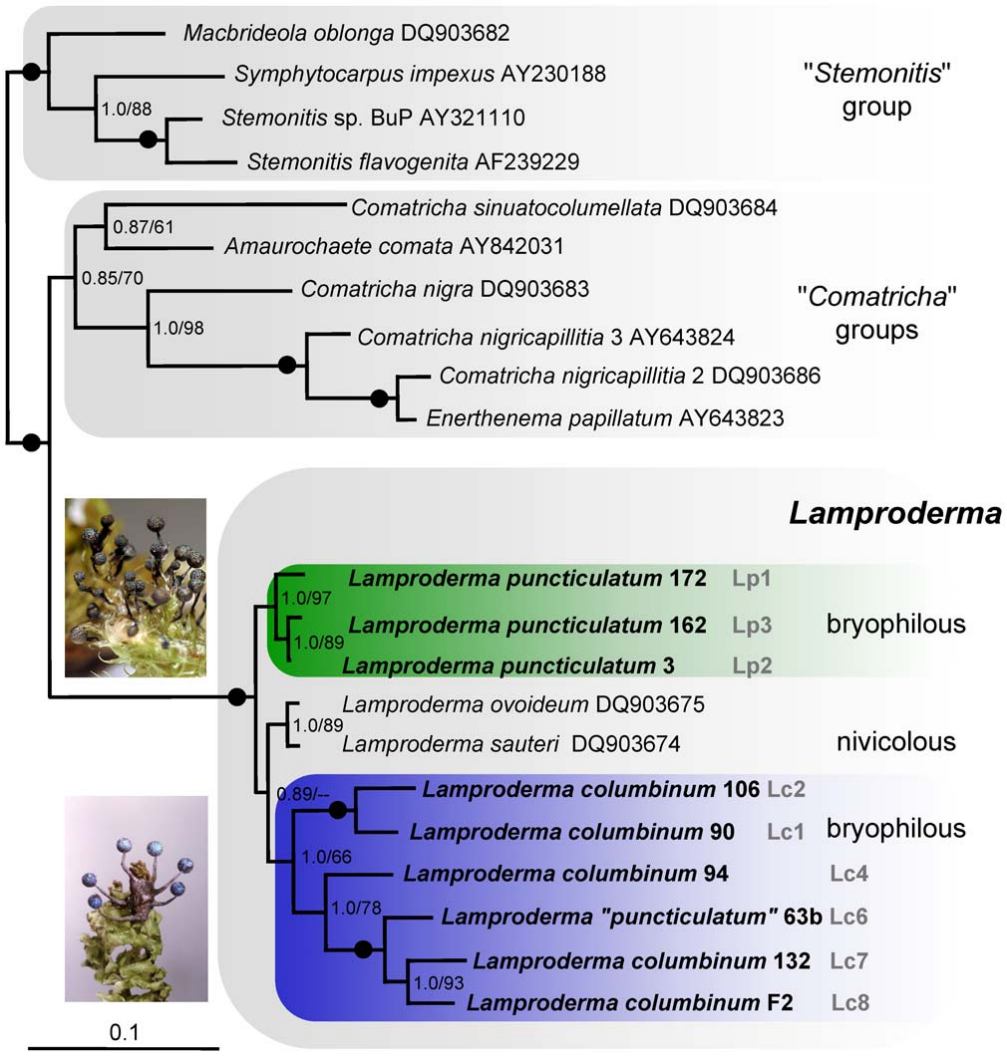
Small subunit (SSU) rRNA gene tree of 21 representatives of Stemonitida. Tree derived by Bayesian inference and Maximum Likelihood analysis of 1598 nucleotide positions. The tree is rooted with the “*Stemonitis*” group according to current phylogenies. Results of one million generation Bayesian posterior probabilities/1000 ML bootstrap replicates are shown for each node. A dot on the line indicates maximum support in both analyses, hyphens indicate bootstrap values <50%. Sequences in bold were obtained during this study. The scale bar indicates the fraction of substitutions per site. Major monophyletic groups are highlighted. Specimens are named and numbered according to Table 2. Genotypes are named Lp1–Lp3 and Lc1–Lc8 as in Fig. 2A.

Based on nearly complete SSU sequences, both Maximum Likelihood and Bayesian analyses resulted in the same tree, robust and well supported even in its basal branches (Fig. 1). The topology of the “*Stemonitis*” and “*Comatricha*” groups was identical to current phylogenies of Myxogastria [31], [39]. All *Lamproderma* specimens formed a monophyletic clade (maximum support in all analyses). The bryophilous *Lamproderma* sequences clearly belonged to two distinct clades, separated by two nivicolous species. Each clade showed a well-supported internal structure (Fig. 1).

### Population genetics of Lamproderma spp

**Figure 2:**
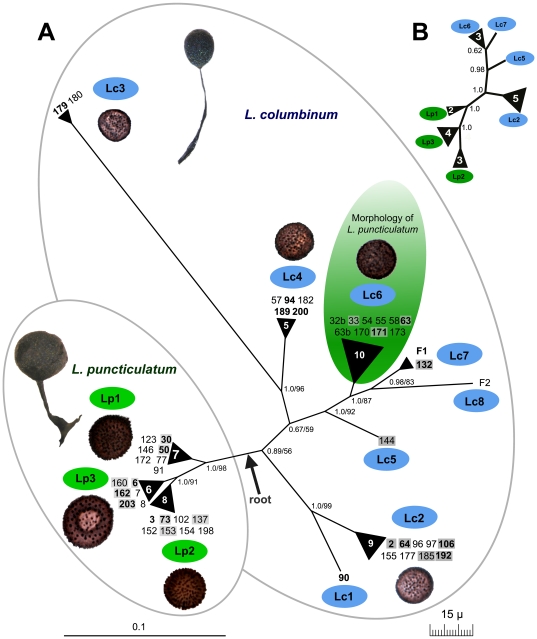
Phylogenies of eleven genotypes of *Lamproderma columbinum* (Lc1–Lc8) and *L. puncticulatum* (Lp1–Lp3). **A: SSU gene** unrooted tree derived by Bayesian inference and Maximum Likelihood analysis of 498 nucleotide positions of the unique 11 genotypes found in the 52 analyzed specimens. Genotypes represented by more than one sequence are indicated by triangles. Specimen numbers for which an ITS and/or an EF-1α sequence was obtained are indicated in bold or shaded by a gray rectangle, respectively. Spore images obtained by light microscopy are reproduced to scale (lower right). The root position (see Fig. 1) is indicated by an arrow, and the resulting two major groups are encircled. Results of one million generation Bayesian posterior probabilities/1000 ML bootstrap replicates are shown for each node. **B: EF-1α** unrooted tree derived by Bayesian inference of 573 nucleotide positions of 19 taxa.

The 52 partial SSU sequences could be grouped into 11 unique genotypes (Fig. 2A). These genotypes also possessed perfectly identical ITS 1 and EF-1α sequences (Fig. 2B). The only exception was found between two groups of specimens, 94, 200 and 57, 182, 189: although the ITS 1 were identical, the SSUs differed slightly by two single-point mutations in two different variable helices. One mutation was neutral (the strength of the pairing with the other strand of the helix is not affected) and the second mutation was an insertion of an extra G in a poly-G – not compensated by an extra C on the other strand. We lumped all five specimens into one genotype (Lc4) because we interpreted these differences as a result of very recent mutations. Bayesian and Maximum likelihood analyses of the partial SSU gave the same tree topology (Fig. 2A), showing an internal genetic structure congruent with the tree obtained with the complete SSU sequences (Fig. 1), and with the EF-1α tree (Fig. 2B). Most branches were well supported, except the two most basal in the *Lamproderma columbinum* clade, i.e. the branch leading to Lc1+Lc2 (posterior probability/bootstrap: 0.89/56) and the one leading to Lc3+Lc4 (0.67/59). When the very divergent sample 179 (Lc3) was removed, the topology remained unchanged, but the basal branches received an improved posterior probability (branch Lc1+Lc2: 0.96, branch Lc4: 1.0), suggesting that the long branch of Lc3 had a destabilizing effect (results not shown).

The specimens of the *Lamproderma columbinum* clade were divided into eight genotypes (Lc1 to Lc8), of which three were singletons. However, specimens of the genotype Lc6 were initially determined as belonging to the *L. puncticulatum* group, displaying smaller spores and less developed warts (Fig. 2A and SEM pictures of specimens 32b = sc21802 and 63 = sc21833 in [32]). It is noteworthy that the SSU sequences of the specimens *L. columbinum* 132 from Saxony and *L. columbinum* F1 from Haute-Savoie (France) were identical, forming the clade Lc7 and were related to the clade Lc8 represented by *L. columbinum* F2 from Savoie (France). The ITS 1 sequences of the clade Lc7 were also identical but for a small difference: an extra 5-bases repeat in a microsatellite-like region in *L. columbinum* F1 (Supporting information S8).

Some genotypes could be characterized by at least two morphological characters: spore size and ornamentation. This was evident for the three genotypes of *L. puncticulatum* (Lp1 to Lp3): whereas all specimens had large spores (15.0–17.5 µm in diameter), there were striking differences between the three genotypes, each being characterized by quite different spore ornamentation (Fig. 2A and SEM pictures of specimens 30 = sc21800 and 6 = sc21779 in [32]). All common genotypes (>2 specimens) were found in both years of collection, 2007 and 2009 (Table 2).

### Statistical evaluation of morphological characters

**Figure 3:**
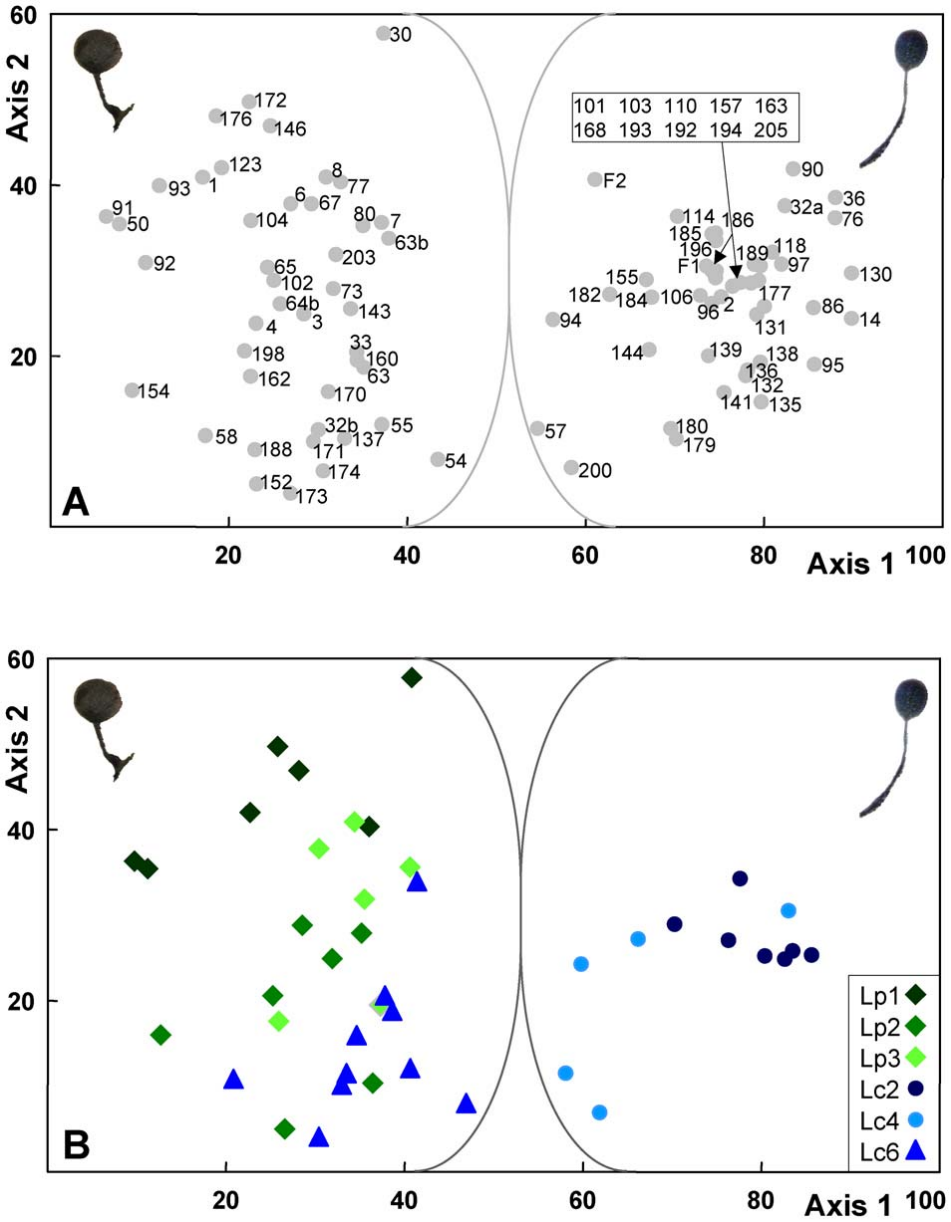
Detrended Correspondence Analysis of 14 morphological traits of specimens of *Lamproderma columbinum* and *L. puncticulatum*. Figure 3A. Plot of 91 specimens collected in Saxony plus two collections from France. Specimen labels refer to Table 2. The left cluster of specimens displays the morphology of *L. columbinum*; the right was determined as *L. puncticulatum*. Figure 3B. Comparison of sample positions in DCA with the six most common genotypes obtained for 45 samples. Note the position of the specimens belonging to *L. columbinum* genotype Lc6 within the cluster of *L. puncticulatum*.

Both PCA (results not shown) and DCA of 14 characters clearly separated the 93 analyzed specimens of *Lamproderma* spp. (91 from Saxony in 2007 and 2009, two from France, Fig. 3A) into two well circumscribed clusters in trait space confirming the results of the previous study in this region [32]. As expected, DCA allowed a better separation of genotypes regardless of the numbers of segments. For a DCA with 26 segments (Fig. 3A) eigenvalues for the first two axes were 0.078 and 0.014, respectively. Except for the genotype Lc6, these clusters were identical to the two major clades obtained in the trees of complete (Fig. 1) and partial (Fig. 2A) SSU sequences. These two groups were also recovered when only the 52 sequenced specimens were considered (results not shown, but nearly identical to Fig. 3B). The two clades differed in sporocarp features, peridial color and capillitium appearance. The latter characters explained most of the variation among samples and were all highly correlated with axis 1 (capillitium diameter 0.915, maximum diameter 0.852, color −0.900, origin −0.815), followed by shape characters (sporotheca shape −0.807, relation stalk to sporotheca −0.778, stalk length −0.760). Peridial color was more variable (0.577). Considering only a selection of characters did not allow for a better separation of the genotype Lc6 from *L. puncticulatum* (results not shown), nor did it improve the distinction of the two clusters coinciding with the major genetic clades.

Typical specimens of *L. columbinum* developed slender, long-stalked sporocarps (stalk 1.8–2.5 times longer than sporotheca) with an ovoid sporotheca and a blue iridescent peridium; the capillitium always consisted of thin, homogenous dark-brown and flexible threads arising from the whole length of the columella. Spore diameter in *L. columbinum* differed significantly between clades: Lc1, Lc2, Lc3, Lc7 and Lc8 had smaller spores (mean 11.8 µm) than clades Lc4 (14.1 µm) and Lc6 (14.7 µm), but all shared an ornamentation consisting of short (mean height 0.5 µm) and rather dense spines. Specimens in the *L. puncticulatum* clade had a more stocky shape, with robust stalks, 1–1.5 (–2) time longer than sporotheca; the sporotheca was spherical and possessed a dark silvery peridium. Capillitial threads were pale, conspicuously enlarged (mean width 8.1 µm in primary branches) and arose from the tip of the columella. Spores were large (mean diameter 16 µm) with a striking ornamentation of tall (mean height 0.8 µm) and scattered warts.

To test for morphological differences in the main genotypes (>2 specimens) we indicated their position in DCA trait space (Fig. 3B). Each genotype was characterized by a set of traits, as they tended to form discrete clusters in the trait space. However, due to considerable overlaps between clusters it was impossible to select an unique set of characters to determine each genotype. It is noteworthy that the group assigned to *L. puncticulatum* with smaller spores was indeed a *L. columbinum*, the clone Lc6. This clone had the overall appearance of *L. puncticulatum*, i.e. a stout sporocarp with spherical sporotheca, although well-matured sprorothecae have a blue iridescent peridium as in *L. columbinum*. The microscopic characters were very confusing, since the mean width of the capillitium was identical to *L. puncticulatum* (8.1 µm), but the spores were of intermediate size between *L. columbinum* and *L. puncticulatum* (mean 14.7 µm) while their ornamentation of small and dense spines was similar to that of *L. columbinum*.

### Mantel test

The Mantel test returned a correlation coefficient of 0.0036 between geographic and genetic distances, well within the 95% confidence interval (−0.0731 to 0.1065) obtained for 999 permutations (mean correlation 0.0016). This clearly indicated an absence of correlation between the geographical and genetic distances. Indeed, most common genotypes were widespread: the genotype Lc2 (9 specimens) was found in seven different localities and the three genotypes of *L. puncticulatum* were found in three (Lp3), four (Lp1) and five localities (Lp2), respectively (Table 2). The only major genotype that was found in a single locality is the *L. columbinum* Lc6 from the Uttewalder Grund (locality 5, Table 2).

### Introns

Group I introns accounted for 57 to 67% of the total length of SSU sequences. Nine insertion sites had been identified in Myxogastria [52] and we have found introns in each of them. Each sequence possessed four to eight introns, 317–1380 bp in length (Supporting information S6), sometimes with microsatellite repeats (e.g. *L. columbinum* 132: introns S911 and S956 and *L. columbinum* F2: intron S1199).

The EF-1α gene obligatory intron in Myxogastria varied in length from 80–81 in *L. columbinum*, with the exception of Lc2, and from 82 to 84 nucleotides in *L. puncticulatum*. The Lc2 intron was 95 nucleotides long due to the insertion of a small microsatellite motif (six TC repeats, see Supporting information S9). Genetic variability was higher in *L. columbinum* (90% similarity) as in *L. puncticulatum* (98%). We did not find any evidence of multiple copies as double-peaks in the chromatograms, excluding the presence of multiple alleles of this gene in all investigated specimens (Supporting information S9).

## Discussion

### Reproductive mode in wild populations of Myxogastria

To assess the genetic structure of *Lamproderma* populations collected in various ravines of the Saxonian Switzerland region, we first targeted the longest fragment of the SSU rRNA devoid of intron insertion sites, including regions that are extremely variable in both sequence and length. Since myxogastrian sequences are characterized by extremely divergent ribosomal sequences [14], [15], we were surprised to find several groups of identical sequences. However, protists with identical SSU rRNA genes can exhibit a high level of genetic divergence if more variable markers, such as the internal transcribed spacer, are used [53], [54]. ITS 1 sequences of related species of *Lamproderma* were found to be so divergent that they were not alignable [10]. Suspecting hidden genetic diversity in our samples, we amplified the ITS 1 locus from several samples of each set of identical SSU sequences. Surprisingly, this did not reveal more variability: specimens with identical SSU sequences had also identical ITS 1, although the ITS 1 sequences were not alignable between clades because of length variation.

Since the ribosomal cistrons are homogeneous and thus not suitable to test for recombination, we targeted the EF-1α gene as a third marker. Again, the EF-1α genes obtained from several specimens of each genotype were identical to each other but differed between genotypes, thus further suggesting that these genotypes may represent asexual clones. Moreover, the chromatograms of the sequences did not reveal the presence of distinct alleles, suggesting that these specimens are all homozygous (or haploid, see Table 1). Heterozygosity can be revealed in the EF-1α chromatograms by double-peaks in the third positions and in the obligatory intron, a pattern observed in the sexual myxogastrian *Echinostelium arboreum* (own unpublished observations). A definitive proof for the existence of asexual clones could be brought by a combination of multiple genetic markers and direct observation of changes in ploidy during the life cycle. Easy to access fingerprinting methods like RAPD or AFLP that require pure DNAs cannot be implemented on field collected sporocarps, thus microsatellite markers need to be established. Obtaining pure DNAs from culture cannot be envisaged as a routine technique for many Myxogastria, like the investigated *Lamproderma* species, as they are notoriously difficult to cultivate. Our data represent a first attempt to investigate the reproductive mode of two *Lamproderma* species, based on only two unrelated genetic markers, and suggest that asexual reproduction may occur in the wild.

### The morphospecies concept may be misleading but is still useful…

By combining genetic analyses with statistical evaluation of morphological characters, we could confirm that *Lamproderma columbinum* and *L. puncticulatum* were indeed distinct genetic entities and that there were only few meaningful characters to allow their recognition, Those characters were mainly capillitium width and stalk length in relation with sporotheca shape, and peridium color, but only in perfectly developed sporocarps. However, the morphological species concept is challenged by two facts. Firstly, spore ornamentation, considered to be one of the most meaningful trait for species differentiation is highly variable between clones, especially in those of *L. puncticulatum*. Secondly, the clone *Lamproderma columbinum* Lc6 displays the characteristics of *L. puncticulatum*, although with some intermediate characters – we first thought it was a *L. puncticulatum* with smaller spores. Without using molecular data for species circumscription it would be difficult to distinguish diverging clones in both species – certainly not welcome news for field ecologists. Species determination based on morphology only is also hampered by a high variability in trait expression, that our statistical analysis has detected even within genotypes, most probably attributable to micro-environmental effects during sporocarp formation (Supporting information S4). On the other hand, the studied species may be seen as collective species with each genotype – or closely related genotypes – representing a separate species. For the investigated *Lamproderma puncticulatum* complex we reject this possibility since our phylogenetic analyses show only a limited genetic distance: variable helices are of identical size (variable region I is of 34 bp); ITS 1 sequences are comparable (620 to 667 bp) and can be aligned (Supporting information S8); and the EF-1α intron shows only seven variable positions (length: 82–84 bp). Because we could obtain only amplifiable DNA from Saxonian specimens and not from the few rare collections of *L. puncticulatum* found elsewhere – and because their genetic structure suggests a recent separation – it would be wiser to modify the species description in order to encompass the encountered morphological variability. This may apply also for the *L. columbinum* Lc6. This genotype, restricted to one locality, may extend its range or disappear in a few years. For *L. columbinum*, where we found rather large genetic distances between genotypes, the description of several separate species could be more justified. Sampling from other localities – this species is cosmopolitan – and another genetic marker would be necessary to answer this question. Summarizing, a modified morphological species concept in Myxogastria should take into account the existence of morphologically diverging clones. Describing each of these clones as a new species seems a titanic task, cluttering up databases and keys. In addition, environmental conditions during sporocarp development unpredictably influence morphology, as found in this study for *L. puncticulatum* forms that have been described as *L. granulosum*.

### Is there any biogeographical pattern?

No correlation between geographical and genetic distance could be retrieved by the Mantel test, and accordingly most genotypes were found in several localities. This indicates that the huge amount of produced spores, estimated between 10^5^ and 10^6^ per sporocarp [55], with usually 10^2^ and 10^3^ sporocarps per colony, is an efficient way of dispersal. Spores may be carried over large distances, as indicated for the genotype Lc7, present in Saxony and in France. In Saxony, the unsuitable habitats separating the ravines – up to 20 km – do not seem to constitute an obstacle. According to a general colonization model for spore-dispersed organisms [55], one should expect short-term advantages for asexually reproducing populations in an ecosystem made of island habitats and our results seem to confirm this model. In previous studies of the reproductive system of populations of *Didymium iridis*, it has been suggested that asexual and sexual strains are sympatric in Central America [27].

Molecular investigations of North-American populations (microsatellites and wide-genome SNPs) of the cellular slime mold *Dictyostelium discoideum* revealed multiple coexisting haplotypes and no geographic structure [56], [57]. In addition recombination, and therefore sexual reproduction was evidenced [56]. This is very surprising since sex, that culminates in a multiwalled immobile macrocyst, was considered a rare event in this taxon [58]. In contrast to myxogastrians, dictyostelids produce their sporocarps by the aggregation of individual amoebae. In *Dictyostelium discoideum*, it has been hypothesized that the coexistence of related strains will favor the evolution of mechanisms enhancing kin recognition which in turn makes it more difficult for opportunistic non-related amoebae to take part in aggregation and form chimeras [56]. Eventually, this should promote sympatric speciation. Thinking along these lines, sympatric instead of allopatric speciation seems the most probable way of genetic isolation in the *Lamproderma* spp. studied herein.

### Conclusions

Molecular genetics of myxogastrian populations is a promising and new field of research. The combination of their being sexual and asexual and having distinctive macroscopic fruiting bodies that have been collected for two centuries from all over the world offers unique advantages over any other group of protists for researching many basic questions in biodiversity, biogeography and speciation. We provide a population-level analysis that shows distinct genetic patterns, that can be interpreted in the light of sexual versus asexual reproduction. Although we cannot deliver ultimate proof for asexual reproduction yet, the rather uniform morphology of some genotypes as well as their sequence identity – in otherwise extremely variable unrelated markers – provides some evidence for it. To study barriers to gene flow in populations that are not yet completely reproductively isolated, we are developing microsatellite markers, taking advantage of one myxogastrian genome in completion and two EST projects. Combining this method with detection of changes in ploidy states during the life cycle should give definite proof for the way myxogastrian species reproduce. In addition, this study lends further evidence to successful long-distance spores dispersal in these protists. The distribution of the genotypes in the limited but highly fragmented landscape studied by us suggests that allopatric speciation may not be the way myxogastrian species arise.

## Supporting Information

Supporting information S1 WebPage showing distribution maps and photos of *L. columbinum*. Available at “The Eumycetozoan project” at the University of Arkansas, http://slimemold.uark.edu/databaseframe.htm (last accessed 10.6.2011).
(PDF)

Supporting information S2 WebPage showing distribution maps and photos of *L. puncticulatum*. Available on the Internet: see S1.
(PDF)

Supporting information S3 WebPage showing distribution maps and photos of *L. granulosum*. Available on the Internet: see S1.
(PDF)

Supporting information S4 Character states of 14 morphological traits recorded for 93 specimens of *Lamproderma* spp. Traits were either measured (m) or estimated according to a scale (s); and observed on three well developed sporocarps of average size (+) or by looking over the whole collection (++). Characters included in the analysis were (1 *Spt_sha*) Sporotheca shape (s++; 1 = depressed, 2 = slightly depressed, 3 = round, 4 = ovate, 5 = ellipsoid); (2 *Spt_wid*; mm) sporotheca width (m+) and (3 *Spt_len*; mm) height (m+); (4 *Cap_min*; µm) minimum and (5 *Cap_max*; µm) maximum capillitium diameter, measured on first-order threads between origin and first branching (m+); (6 *Cap_dia*) capillitium shape (s++; 1 = thread-like, 2 = slightly flattened, 3 = clearly band-like flattened) and (7 *Cap_col*) color under transmitted light (s+; 1 = nearly colorless, 2 = pale brown, 3 = brown); (8 *Cap_ori*) capillitium origin (s+; 1 = on top of columella only, 2 = somewhat decurrent, 3 = from the whole length of columella); (9 *Per_col*) peridium color (s++; 1 = iridescent blue, 2 = mixed, 3 = dull silvery); (10 *Sta/Spt*) ratio stalk length to sporotheca height (s++); (11 *Sta_len*; mm) stalk length (m+); (12 *Sp_dia*; µm) spore diameter (m+; 15–25 spores per sporocarp); (13 *Spi_len*; µm) length of spore ornaments (spinulae, m+; 5–10 spores per sporocarp) and (14 *Spi_den*) density of spore ornaments (m+; spinulae per 9 µm^2^ spore surface, 3 spores per sporocarp).
(XLS)

Supporting information S5 Location of the Elbe Sandstone Mountains and places of collection of the specimens used in this study, indicated by disks. Adjacent localities are plotted together, identified by numbers as in Table 2. The diameter of the disks is proportional to the number of collected specimens.
(PDF)

Supporting information S6 Alignment in fasta format related to the tree in Fig. 1, including 21 representatives of Stemonitida. The first sequence is the mask indicating the positions retained for analysis. The last sequence indicates an approximate secondary structure of some variables helices.
(FAS)

Supporting information S7 Alignment in fasta format related to the tree in Fig. 2A: Partial (first ca. 600 bp) of the SSU of the 12 unique sequences of *Lamproderma* spp. obtained in this study. The first sequence is the mask indicating the positions retained for analysis.
(FAS)

Supporting information S8 Alignment in fasta format of the ten unique ITS 1 sequences, partially aligned.
(FAS)

Supporting information S9 Alignment in fasta format of 19 EF-1α sequences (related to the tree in Fig. 2B), along with most of hitherto available myxogastrian sequences. Only the ca. first 1000 positions are shown. The intron obligatory for myxogastrians starts at position 692.
(FAS)
