# Supplementary material for: Genetic Structure of Two Protist Species (Myxogastria, Amoebozoa) Suggests Asexual Reproduction in Sexual Amoebae
Source: PLoS One. 2011 Aug 1;6(8):e22872. doi: 10.1371/journal.pone.0022872 (PMC3148230; doi:10.1371/journal.pone.0022872)
Supplement: Supporting information S1 — WebPage showing distribution maps and photos of L. columbinum. Available at “The Eumycetozoan project” at the University of Arkansas, http://slimemold.uark.edu/databaseframe.htm (last accessed 10.6.2011). (PDF) [file pone.0022872.s001.pdf]

[The Eumycetozoan Project](#) | [Search](#) | [All Living Things](#)

Lamproderma columbinum **(Pers) Rostaf**
Life Amoebozoa Eumycetozoa
Stemonitidaceae Lamproderma

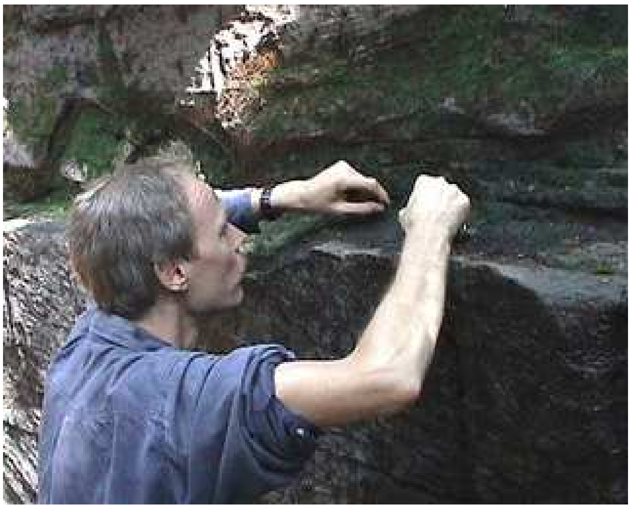

© The Eumycetozoan Project, 2006
Lamproderma columbinum

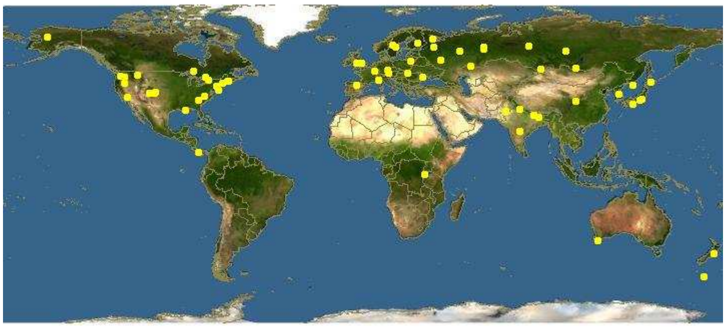

Click on map for details about points.

IDnature guides

- Mycetozoa GSMNP
- Myxomycetes

80x5 - 240x3 - 240x4 - 320x1 - **320x2** - 320x3 - 640x1 - 640x2
Set display option above. Click on images to enlarge.

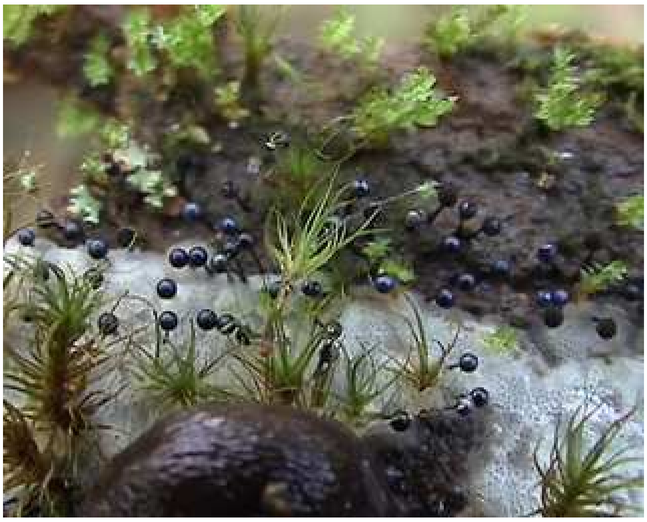

© The Eumycetozoan Project, 2006
Lamproderma columbinum

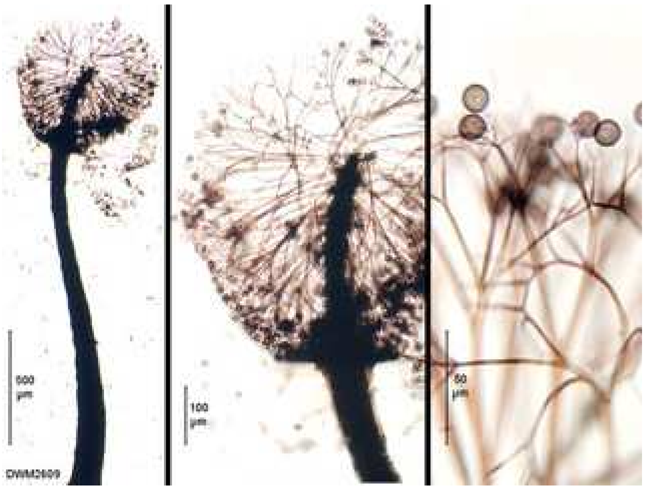

© The Eumycetozoan Project, 2006
Lamproderma columbinum

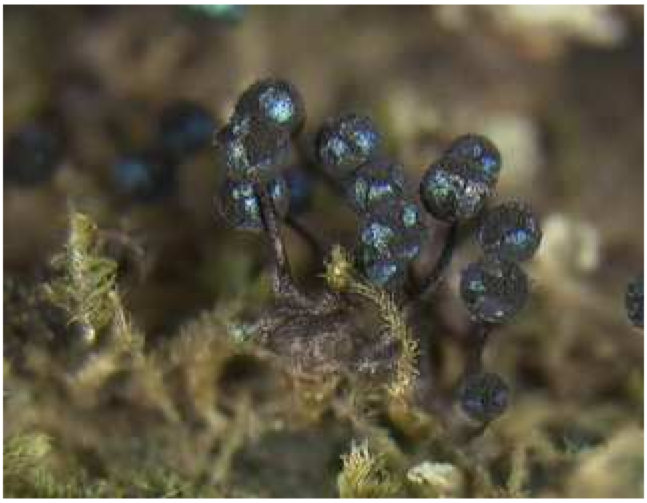

© The Eumycetozoan Project, 2006

Lamproderma columbinum

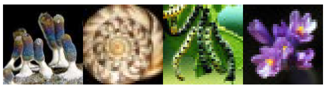

4 thumbnails • slide show

Overview

Sporocarps scattered or in groups, long-stalked, up to 5 mm total height. Sporothecae globose or ellipsoid, 0.5-1 mm diam., blue with violet and purple iridescence. Hypothallus dark purple. Stalk black, 75% of the total height. Columella cylindrical with a blunt apex, reaching the centre of the sporotheca. Capillitium purple-brown, arising from the length of the columella, rigid, branched and outwardly anastomosed to form an open net. Spore-mass black. Spores grey-brown, 10-13 µm diam., verruculose. Plasmodium white.

Names

Scientific source:

- Integrated Taxonomic Information System

Links to other sites

- Searchable databases -- The Eumycetozoan Project

References

- Ing,B. 1983: A ravine association of Myxomycetes. *Journal of biogeography 10*: 299-306.
- Lister,G. 1923: Lamproderma columbinum Rost. and its varieties. *Transactions of the British Mycological Society 9*: 32-34.
- Roth,J.-J. 1995: Der Pilz des Monats: Lamproderma columbinum (Pers.) Rost.. *Schweizerische Zeitschrift für Pilzkunde 11*: 209-210.

Acknowledgements

The Eumycetozoan Project -- working to understand the ecology, sytematics and evolution of myxomycetes, dictostelids and protostelids -- the true slime molds.

Sponsored by grants from the National Science Foundation.

Feedback

Please send any corrections and comments about this page to John Shadwick
Department of Biological Sciences, University of Arkansas, Fayetteville, AR 72701, USA
email: jshadwi@uark.edu phone: USA-479-575-7393.

Supported by

- National Biological Information Infrastructure
- National Science Foundation

Updated: 2011-06-16 21:58:37 gmt

© Designed by The Polistes Corporation
