# Supplementary material for: Genetic Structure of Two Protist Species (Myxogastria, Amoebozoa) Suggests Asexual Reproduction in Sexual Amoebae
Source: PLoS One. 2011 Aug 1;6(8):e22872. doi: 10.1371/journal.pone.0022872 (PMC3148230; doi:10.1371/journal.pone.0022872)
Supplement: Supporting information S2 — WebPage showing distribution maps and photos of L. puncticulatum. Available on the Internet: see S1. (PDF) [file pone.0022872.s002.pdf]

The Eumycetozoan Project | Search | All Living Things

Lamproderma puncticulatum **Härk**
Life Amoebozoa Eumycetozoa
Stemonitidaceae Lamproderma

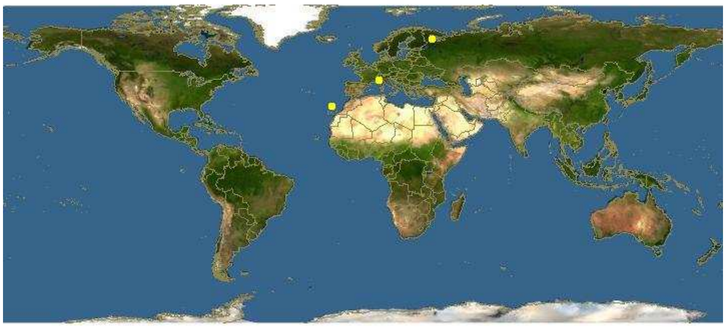

Click on map for details about points.

IDnature guide

- Myxomycetes

Overview
Sporocarps gregarious, short-stipitate, 0.5-1.3 mm total height. Sporotheca dark silvery-grey, globose, 0.4-0.8 mm diam. Peridium membranous, rough, persistent, iridescent with blue, gold, green and red reflections and with small, dark brown, non-iridescent depressions. Stalk either absent or up to 0.5 mm tall, thick, furrowed and concolorous with the sporotheca. Hypothallus stout, discoid, concolorous with the sporotheca and partly confluent. Columella black, robust, cylindrical, c. 30-50% of the sporotheca height. Capillitium arising from the columella apex, pale brown throughout, branched and anastomosed and with many free ends, the threads 3-8 µm diam. at the base, less than 3 µm at the periphery. Spore-mass dark brown. Spores purple-brown, spinulose, 12.5-14 µm diam.

Links to other sites

- Searchable databases -- The Eumycetozoan Project

References

- Härkönen,M. 1978: Lamproderma puncticulatum, a new species of Myxomycetes. *Karstenia 18*: 20-22.

Acknowledgements

The Eumycetozoan Project -- working to understand the ecology, sytematics and evolution of myxomycetes, dictostelids and protostelids -- the true slime molds.

Sponsored by grants from the National Science Foundation.

Feedback

Please send any corrections and comments about this page to John Shadwick
Department of Biological Sciences, University of Arkansas, Fayetteville, AR 72701, USA
email: jshadwi@uark.edu phone: USA-479-575-7393.

Supported by

- National Biological Information Infrastructure
- National Science Foundation

Updated: 2011-06-17 07:13:09 gmt

© Designed by The Polistes Corporation
