# Supplementary material for: Genetic Structure of Two Protist Species (Myxogastria, Amoebozoa) Suggests Asexual Reproduction in Sexual Amoebae
Source: PLoS One. 2011 Aug 1;6(8):e22872. doi: 10.1371/journal.pone.0022872 (PMC3148230; doi:10.1371/journal.pone.0022872)
Supplement: Supporting information S3 — WebPage showing distribution maps and photos of L. granulosum. Available on the Internet: see S1. (PDF) [file pone.0022872.s003.pdf]

Lamproderma granulosum N. H Neubert, owotny et Schnittler

Life Amoebozoa Eumycetozoa Stemonitidaceae Lamproderma

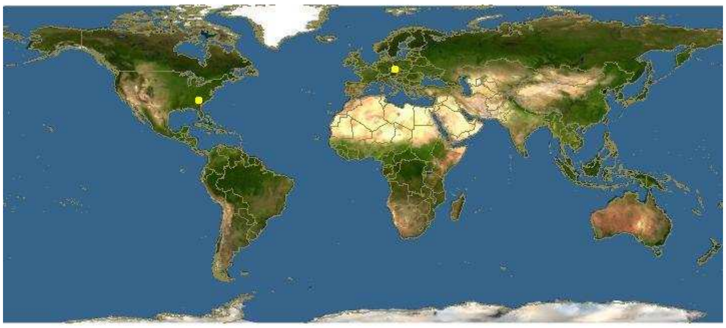

Click on map for details about points.

IDnature guide

- Myxomycetes

Overview

Sporocarps scattered or grouped, up to 2 mm total height. Sporotheca globose or ovoid on a wide base, 0.4-0.8 mm diam., up to 0.8 mm high, dark greyish brown and slightly blue-violet iridescent. Stalk opaque, black, 0.5-1.5 mm tall, longitudinally wrinkled, basally expanded into the hypothallus. Peridium persistent, the surface punctate, grey-brown to black, the base wrinkled and darker, the upper region punctate, hyaline to brown and marbled. Columella black, cylindrical, reaching to c. the centre of the sporotheca. Capillitium radiating from the upper half of the columella, rigid, flattened, 6-10 µm diam., tapered and pale at each end, laxly branched and anastomosed, containing 0.5-4 µm diam. globules similar to those in Cribraria. Hypothallus membranous, dark brown, discoid to individual sporocarps. Spore-mass black. Spores dark grey-brown, 15-19 µm diam., irregularly spinose, the spines 0.5-1.5 µm tall. Plasmodium watery white. On liverworts.

Links to other sites

- Searchable databases -- The Eumycetozoan Project

References

- Neubert,H., Nowotny, W., Schnittler, M. 1990: Myxomyceten aus der Bundesrepublik Deutschland IV. Lamproderma granulosum sp. nov., eine neue Art aus dem Elbsandsteingebirge. *Beiträge zur Kenntnis der Pilze Mitteleuropas 6*: 49-52.

Acknowledgements

The Eumycetozoan Project -- working to understand the ecology, sytematics and evolution of myxomycetes, dictostelids and protostelids -- the true slime molds.

Sponsored by grants from the National Science Foundation.

Feedback
Please send any corrections and comments about this page to John Shadwick
Department of Biological Sciences, University of Arkansas, Fayetteville, AR 72701, USA
email: jshadwi@uark.edu   phone: USA-479-575-7393.

Supported by

- National Biological Information Infrastructure
- National Science Foundation

Updated: 2011-06-17 07:14:33 gmt

The Eumycetozoan Project | Search | All Living Things | Top

© Designed by The Polistes Corporation
