# Supplementary material for: Genetic Structure of Two Protist Species (Myxogastria, Amoebozoa) Suggests Asexual Reproduction in Sexual Amoebae
Source: PLoS One. 2011 Aug 1;6(8):e22872. doi: 10.1371/journal.pone.0022872 (PMC3148230; doi:10.1371/journal.pone.0022872)
Supplement: Supporting information S5 — Location of the Elbe Sandstone Mountains and places of collection of the specimens used in this study, indicated by disks. Adjacent localities are plotted together, identified by numbers as in Table 2. The diameter of the disks is proportional to the number of collected specimens. (PDF) [file pone.0022872.s005.pdf]

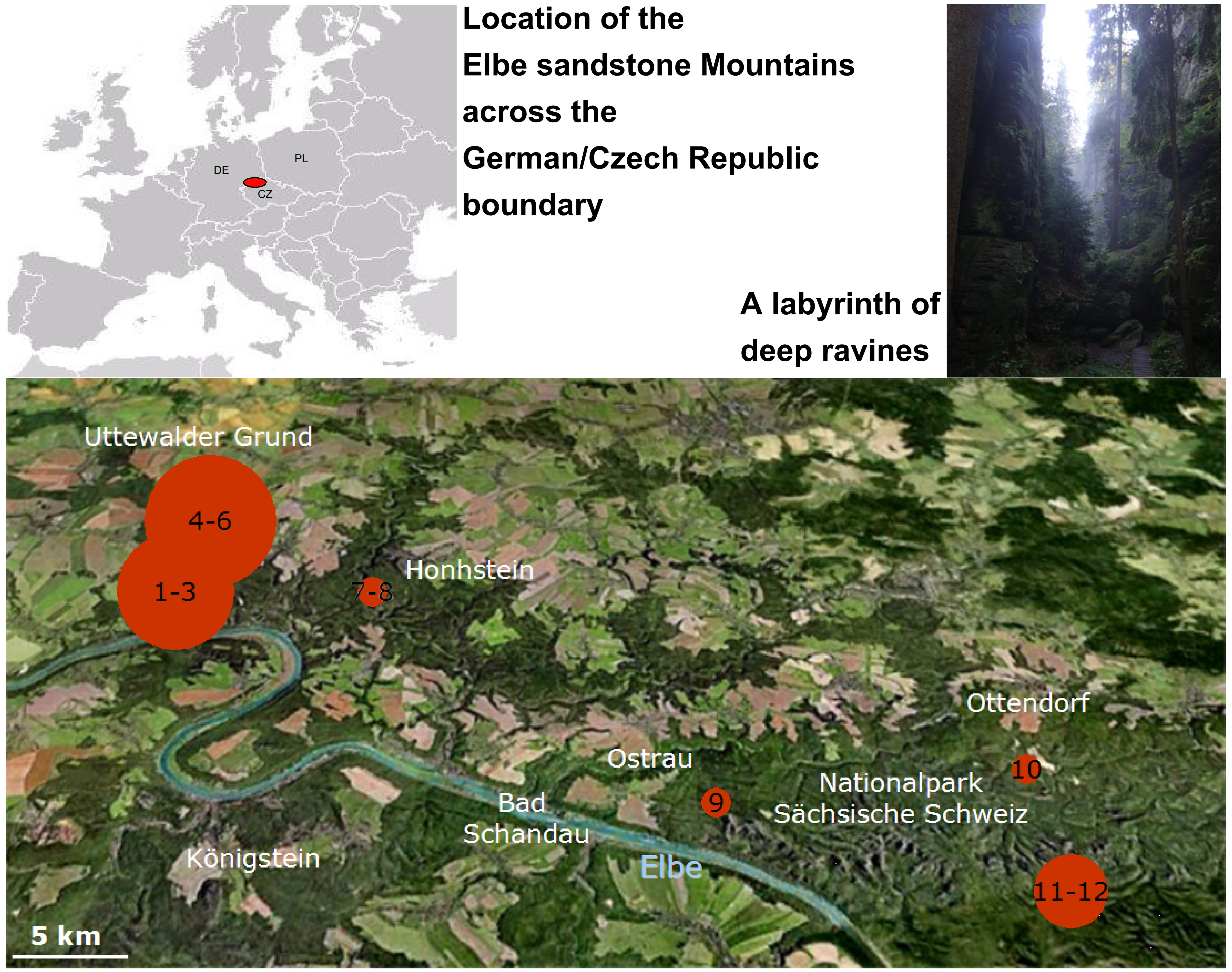

Places of collection of the specimens used in this study, indicated by disks. Adjacent localities are plotted together, identified by numbers as in Table 2. The diameter of the disks is proportional to the number of collected specimens.
